# The effect of exercise training and motivational counselling on physical activity behaviour and psychosocial factors in pregnant women: secondary analyses of the FitMum randomised controlled trial investigating prenatal physical activity

**DOI:** 10.1186/s12889-023-17525-3

**Published:** 2024-01-04

**Authors:** Signe de Place Knudsen, Caroline Borup Roland, Saud Abdulaziz Alomairah, Anne Dsane Jessen, Helle Terkildsen Maindal, Jane M. Bendix, Tine D. Clausen, Ellen Løkkegaard, Bente Stallknecht, Stig Molsted

**Affiliations:** 1 Department of Biomedical Sciences, University of Copenhagen, Copenhagen, Denmark; 2 Department of Gynaecology and Obstetrics, Copenhagen University Hospital - North Zealand, Hillerod, Denmark; 3 Public Health Department, Saudi Electronic University, College of Health Sciences, Riyadh, Saudi Arabia; 4 Department of Public Health, Aarhus University, Aarhus, Denmark; 5 Steno Diabetes Center Copenhagen, Herlev, Denmark; 6 Department of Clinical Research, Copenhagen University Hospital - North Zealand, Hillerod, Denmark; 7 Department of Clinical Medicine, University of Copenhagen, Copenhagen, Denmark

**Keywords:** Maternal exercise interventions, Pregnancy, Physical activity, Behavioural regulation in exercise, Self-efficacy, Health-related quality of life, Sick leave, Low back pain, Pelvic girdle pain, FitMum

## Abstract

**Background:**

A physically active lifestyle is beneficial during pregnancy. However, little is known about physical activity (PA) behaviour and psychosocial factors in women during and after pregnancy. This study examined exercise behavioural regulation, exercise self-efficacy, health-related quality of life, sickness absence and musculoskeletal pain in pregnant women offered either structured supervised exercise training, motivational counselling on PA, or standard prenatal care in the FitMum randomised controlled trial.

**Methods:**

Two hundred and eighteen healthy inactive pregnant women were randomised to structured supervised exercise training (*n* = 87), motivational counselling on PA (*n* = 86) or standard prenatal care (*n* = 45). The women answered the Behavioural Regulation in Exercise Questionnaire-2 (BREQ-2), the Pregnancy Exercise Self-Efficacy Scale (P-ESES-DK) and the Short Form 36 Health Survey Questionnaire (SF-36) at baseline (gestational age (GA) of max 15 weeks), GA 28 and 34 weeks, and one year after delivery. Sickness absence and low back and/or pelvic girdle pain were likewise reported in questionnaires at baseline and GA 28 weeks.

**Results:**

Participants offered structured supervised exercise training or motivational counselling on PA had higher autonomous motivation for exercise during pregnancy compared with participants receiving standard prenatal care (e.g., difference in intrinsic regulation at GA 28 weeks, structured supervised exercise training vs. standard prenatal care: mean difference in score 0.39 [0.16; 0.64], *p* < 0.001). Participants offered structured supervised exercise training also had higher exercise self-efficacy during pregnancy (e.g., GA 28 weeks, structured supervised exercise training vs. standard prenatal care: mean difference in score 6.97 [2.05; 12.02], *p* = 0.005). All participants reported high exercise self-efficacy at baseline and medium exercise self-efficacy during pregnancy and one year after delivery. No differences were found between groups in health-related quality of life, sickness absence or low back and/or pelvic girdle pain during pregnancy. No group differences were found one year after delivery.

**Conclusion:**

Structured supervised exercise training and motivational counselling on PA had important effects on autonomous exercise motivation during pregnancy. Exercise self-efficacy was also increased with structured supervised exercise training compared to standard prenatal care. No group differences in health-related quality of life, sickness absence, or pain were found during and after pregnancy. No effects were found one year post-delivery after intervention cessation.

**Trial registration:**

The study was approved by the Danish National Committee on Health Research Ethics (#H-18011067) and the Danish Data Protection Agency (#P-2019–512). The study adheres to the principles of the Helsinki declaration. Written informed consent was obtained at inclusion.

**Supplementary Information:**

The online version contains supplementary material available at 10.1186/s12889-023-17525-3.

## Background

Pregnant women's compliance with the internationally recommended minimum of 150 min of physical activity (PA) per week remains low despite substantial evidence supporting the benefits of a physically active lifestyle during pregnancy [1–9]. Over the years, pregnancy has been referred to as a time period in which women can be particularly interested in improving their health, as they may be more receptive to health messages and are in frequent contact with health professionals [10–12]. Despite the ‘teachable moment’ [10, 12] or ‘window of opportunities’ [13] and the recognized importance of safe and beneficial PA for both pregnant women and the general population [14], a considerable decline in PA from preconception throughout pregnancy has been observed [1, 15]. Frequently reported barriers towards PA are fatigue, lack of time and pregnancy discomfort such as nausea, pain and awkwardness due to weight gain and increasing size [14].

One approach to understand the initiation and maintenance of health behaviours, including PA, is the Self-Determination Theory [16]. This theory classifies motivation for PA on a spectrum ranging from amotivation with no intention to perform a given behaviour through extrinsic motivation continuing to intrinsic motivation [17, 18]. Extrinsic motivation is characterised by performing an activity based on extrinsic reasons, for achieving certain outcomes or to attain a tangible reward, whereas intrinsic motivation implies performing a task ‘for one’s own sake’ or for inherent interest and enjoyment [16]. Within the context of Self-Determination Theory, it is possible to evaluate a nuanced aspects of various forms of motivation that drive individuals to engage in PA, providing valuable insights for both research and practical applications [19].

In healthy adults, the adoption and maintenance of PA behaviour are associated with exercise self-efficacy [20, 21]. The Self-Efficacy Theory posits a strong link between perceived and actual ability to perform behaviours, including PA [22]. However, owing to the scarcity of studies on the effect of PA interventions on self-efficacy among pregnant women and the inconsistencies of their conclusions, it is challenging to attribute increased PA levels to enhanced self-efficacy. Studies of effects of PA interventions on self-efficacy in maintaining regular exercise routines for pregnant women are therefore needed as recommended in a previous systematic review [23].One potential benefit of maintaining higher levels of PA during pregnancy is an improvement in health-related quality of life (HRQoL). Pregnancy can lead to decreased HRQoL, encompassing physical, mental, and social aspects [24–26]. Challenges that may reduce the HRQoL include musculoskeletal pain, emotional distress, and changes in body image [21, 27]. However, it has been suggested that regular PA during pregnancy can mitigate the negative impact of these challenges on HRQoL [28–30].

Notably, low back and/or pelvic girdle pain are at some point prevalent among most women during pregnancy, affecting pregnant women’s ability to exercise and their overall well-being. The presence of pain often leads to a decreased level of PA, increased sickness absence, and exacerbation of discomfort [31–34]. In light of these considerations, a comprehensive understanding of exercise training’s effect on PA behaviour, musculoskeletal pain, and sickness absence is essential with important implications for public health.

Our research group conducted the FitMum trial, targeting inactive pregnant women to explore the impact of structured supervised exercise training (EXE) or motivational counselling on PA (MOT) on PA levels compared to standard prenatal care (CON) [35]. At inclusion, the median gestational age (GA) was 12.9 (IQR 9.4–13.9) weeks. The average moderate-to-vigorous-intensity PA (minutes per week) from randomisation to GA 28 weeks was 50 [95% confidence interval (CI) 39; 60] in EXE, 40 [95% CI 30; 51] in MOT, and 33 [95% CI 18; 47] in CON. When adjusted for baseline moderate-to-vigorous-intensity PA, participants in EXE performed 20 [95% CI 4; 36] and 21 [95% CI 3; 39] minutes per week more moderate-to-vigorous-intensity PA than participants in CON from randomisation to GA 28 weeks and birth (*p* = 0.02 and *p* = 0.02), respectively. No effect on moderate-to-vigorous-intensity PA was found in MOT compared to CON, and moderate-to-vigorous-intensity PA did not differ significantly between EXE and MOT [36].

Other PA interventions during pregnancy have also shown small yet important effects on increasing PA levels [37]. The present secondary analyses of the FitMum trial sought to evaluate the influence of PA interventions on PA behaviour, exercise self-efficacy, HRQoL, as well as the occurrence of sickness absence and musculoskeletal pain among participating pregnant women.

## Methods

### Participants and study design

Two hundred and twenty healthy, inactive pregnant women were enrolled in FitMum, a randomised controlled single-site trial. The trial was conducted in 2018–2021 at the Department of Gynaecology and Obstetrics at Copenhagen University Hospital – North Zealand, Denmark (ClinicalTrials.gov #NCT03679130) [35]. Participants were defined as inactive if they performed one hour or less of moderate-to-vigorous-intensity structured exercise per week during early pregnancy. The PA level was assessed based on in-depth interviews before inclusion. At inclusion (GA of max 15 weeks), the participants’ demographic data were collected. Before randomisation, one participant withdrew her consent. Thus, 219 participants were randomised to EXE (*n* = 87), MOT (*n* = 87), or CON (*n* = 45) [35]. One participant randomised to MOT withdrew her consent after end of study. Therefore, 218 participants were included in the analyses. In brief, participants in EXE were offered one-hour supervised exercise training at moderate intensity three times/week in a gym and a swimming pool. The MOT intervention consisted of four individual and three group PA motivational counselling sessions of 1–2 h duration during pregnancy, and one weekly personalised text message to support PA. All groups were offered standard prenatal care including consultations with their general practitioner and midwife. During the Covid-19 pandemic (from March 11th, 2020, and throughout the rest of the intervention period) EXE and MOT sessions were adapted into an online format and conducted virtually. The adaptations consisted primarily of the EXE where advanced training equipment were not used. The interventions ran from randomisation to delivery [35, 38].

### Outcome measures

Questionnaire data was electronically reported by the participants using the Danish versions of the Behavioural Regulation in Exercise Questionnaire-2 (BREQ-2) [19], the Pregnancy Exercise Self-Efficacy Scale (P-ESES-DK) [39, 40], and the Short Form 36 Health Survey Questionnaire (SF-36) volume 2 [41, 42] at baseline (GA (gestational age) of max 15 weeks), GA 28 and 34 weeks, and one year after delivery. Sickness absence as well as low back and pelvic girdle pain were reported at baseline and GA 28 weeks.

#### Behavioural regulation in exercise

The Danish version of the 19-item BREQ-2 was used to capture reasons for exercise that vary along a graded continuum of self-determination from amotivation to controlled (external and introjected) and to more autonomous (identified and intrinsic) regulations [19]. The tool comprises five subscales: 1) amotivation (four items, Cronbach’s Alpha 0.83), where a person has no intention to perform PA (e.g., ‘I don’t see why I should have to exercise’), 2) external regulation (four items, Cronbach’s Alpha 0.79), where PA is performed as a result of rewards or punishment given by another person (e.g. ‘I exercise because other people say I should’), 3) introjected regulation (three items, Cronbach’s Alpha 0.80), where PA is performed to increase one's self-esteem or avoid negative emotions (e.g. ‘I feel guilty when I don’t exercise’), 4) identified regulation (four items, Cronbach’s Alpha 0.73), where PA can help a person to achieve goals set (e.g. ‘I value the benefits of exercise’), and 5) intrinsic regulation (four items, Cronbach’s Alpha 0.86), where PA is performed because the person finds it enjoyable in itself (e.g. ‘I exercise because it’s fun’). Autonomous motivation includes intrinsic motivation and the types of extrinsic motivation (introjected and identified) where people have identified themselves with the value of an activity and ideally have integrated it into their self-understanding [43, 44].

Responses to each BREQ-2 item were made on a 5-point Likert scale ranging from ‘0’ (not true) to ‘4’ (very true). The mean scores of the three to four statements related to each of the five types of regulation of exercise behaviour were calculated, giving separate scores for each type of regulation. The multidimensional scoring ranges from 0 to 20 [44, 45].

#### Pregnancy exercise self-efficacy

Changes in self-efficacy of exercise behaviour were assessed using The Danish version of the P-ESES [40]. The P-ESES [46] consists of 10 questions assessing the ability and motivation to complete PA under different circumstances (Cronbach’s Alpha 0.84). The full set of P-ESES items is preceded by the statement: ‘I am confident that I can’ followed by each item, e.g., item 1: ‘Overcome barriers and challenges to exercise if I try hard enough’. Participants were asked to rate their current beliefs in their ability to complete 30 min of exercise each day on a 5-point scale from ‘1’ (strongly disagree) to ‘5’ (strongly agree). The outcome is reported as the sum of the 10 questions ranging from 10 to 50 with a score between 35 and 50 indicating high exercise self-efficacy, 18–34 medium exercise self-efficacy, and 10–17 low exercise self-efficacy [47].

#### Health-related quality of life

The Danish version of the SF-36 was used to identify the participants’ physical, social, and mental health. The tool includes the items physical functioning (Cronbach’s Alpha 0.92), role limitations due to physical function (Cronbach’s Alpha 0.87), bodily pain (Cronbach’s Alpha 0.82), general health perceptions (Cronbach’s Alpha 0.78), vitality (Cronbach’s Alpha 0.85), social function (Cronbach’s Alpha 0.78), emotional function (Cronbach’s Alpha 0.75), and mental health (Cronbach’s Alpha 0.80). The eight items were aggregated into Physical and Mental Component Summary scores derived as the weighted sum of the item scores using the US standard SF-36 scoring algorithms [41, 42]. The two variables were transformed to a score between 0 and 100, where higher score indicates higher HRQoL. The Physical and Mental Component Summary scores were considered the primary SF-36 outcomes of this study.

#### Sickness absence and sick leave

The present study differentiated between sickness absence (without a medical certificate) and sick leave (based on a medical certificate). In Denmark, pregnant women may obtain paid sick leave with a medical certificate issued by their general practitioner. The sick leave may be granted full-time or at varying degrees of part-time. Data was self-reported using a close-ended question (yes/no) on whether sickness absence and/or sick leave from work or study during pregnancy were experienced. If sickness absence and/or sick leave were experienced, the number of days (0–4, 5–8, 9–14, above 14 days) and cause (pregnancy-related, not pregnancy-related (including ‘other reasons’), or due to the work environment) were obtained.

#### Low back and pelvic girdle pain

Low back pain was defined as pain localised between the 12th rib and the inferior gluteal folds with or without leg pain. Pelvic girdle pain was defined as pain experienced between the posterior hip crest and the gluteal fold, particularly near the sacroiliac joints. The pain could radiate into the posterior thigh and occur in association with/or separately in the symphysis [48]. In the present study, the two terms are referred to as musculoskeletal pain. Together with an illustration of a woman marked with the low back and pelvic girdle pain definitions, the history of pain was obtained by questions about how often (daily, weekly, or never) musculoskeletal pain was experienced prior to the current pregnancy (whether the woman habitually experienced pain) and within in the last 14 days, respectively. If musculoskeletal pain was experienced, the women were asked to what extent when experienced least, worst and on average rated from 0 (no pain) to 10 (worst possible pain) on the 11-item numeric rating scale [49].

### Statistical analyses

All statistical analyses were performed using R [50]. Descriptive statistics are presented as mean ± standard deviation (SD) for approximately symmetric distributions, median and interquartile range (IQR) for asymmetric distributions, and frequencies and proportions for categorical data. Estimated effects sizes are presented with [95% CI]. In accordance with the CONSORT (Consolidated Standards of Reporting Trials) guidelines [51], no statistical comparisons have been performed for descriptive analyses.

For BREQ-2, P-ESES-DK and SF-36, a constrained linear mixed model was fitted with the observation times as a factor, and the analyses were performed with bootstrap (*n* = 1000) [52]. Whilst the analyses were not based on the ‘intention to treat’ principles, the linear mixed model takes missing data into account. The numbers of missing data are presented in the Results section. Between-group effects are reported as estimated differences in means. Observations of the questionnaire data throughout the study period are reported descriptively. Sickness absence and sick leave were analysed with Pearson’s chi-squared test or the Fisher’s exact test on small samples in subscales. Pain is presented with medians and IQR, and the non-parametric Kruskal Wallis test was used to test differences between groups. The associations between level of PA and psychosocial factors were tested using linear regression analyses. In the regression analyses, moderate-to-vigorous-intensity PA (min/week) was the exposure as this variable outcome was the primary outcome in a primary effect analysis of the FitMum study, and the outcome variables were the data from GA 28 weeks. Data are presented as β [95% CI]. The level of statistical significance was 5% for all analyses.

## Results

### Characteristics of participants

Maternal baseline characteristics of the 218 participants are presented in Table 1. At inclusion participants were almost 13 weeks of gestation, just over one third were first-time parents, and the majority had more than 12 years of education.

**Table 1:** Baseline characteristics of participants

	**ALL**	**EXE**	**MOT**	**CON**
	***n* = 218**	***n* = 87**	***n* = 86**	***n* = 45**
**Age (years)**	31.5 ± 4.3	31.1 ± 4.3	31.7 ± 4.1	32.0 ± 4.6
**Gestational age at inclusion (weeks)**	12.8 (9.4–13.9)	12.6 (9.3–13.7)	12.8 (9.6–13.9)	12.9 (9.7–13.9)
**Weight (kg)**	75.4 ± 15.4	76.2 ± 17.4	76.4 ± 13.8	72.0 ± 13.7
**Pre-pregnancy BMI (kg/m**^**2**^**)**	24.1 (21.7–28.7)	25.1 (21.5–29.7)	24.1 (22.3–29.0)	23.5 (21.3–26.8)
**Parity, nulliparous**	82 (38%)	40 (46%)	26 (30%)	16 (36%)
**Educational level**				
** School ≥ 12 years**	191 (88%)	74 (85%)	76 (88%)	41 (91%)
** Further education ≥ 3 years**	175 (80%)	73 (74%)	69 (79%)	33 (73%)
** Employed/studying**	198 (91%)	83 (95%)	76 (88%)	39 (87%)

Baseline characteristics of the participants in FitMum. Descriptive data are presented as means ± SD, medians (IQR), or n (%). School ≥ 12 years corresponds to high school. Further education ≥ 3 years corresponds to a university degree (bachelor or master level). *SD* standard deviation, *IQR* interquartile range, *n* number, *BMI* body mass index, *EXE* structured supervised exercise training, *MOT* motivational counselling on physical activity, *CON* standard prenatal care. No statistical comparisons have been performed on descriptive characteristics in accordance with CONSORT guidelines [51]

### Exercise behavioural regulation

**Fig. 1:**
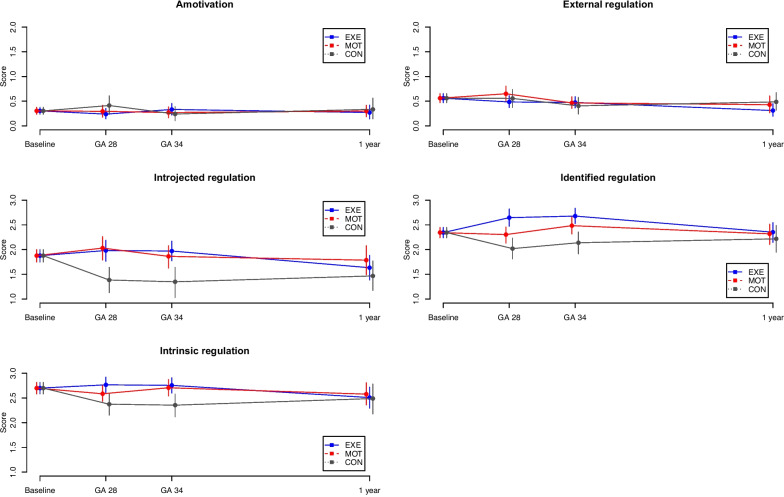
Behavioural Regulation in Exercise Questionnaire-2 subscales Behavioural Regulation in Exercise Questionnaire-2 subscales (mean and 95% CI) measured at baseline, GA 28 weeks, GA 34 weeks and one year after delivery. The graded continuum of self-determination ranges from amotivation to controlled (external and introjected) and to more autonomous (identified and intrinsic) regulations. GA, gestational age in weeks; blue, structured supervised exercise training (EXE); red, motivational counselling on physical activity (MOT); grey, standard prenatal care (CON)

BREQ-2 was completed at baseline, GA 28 weeks, GA 34 weeks, and one year after delivery by 100% (*n* = 218/218), 83.0% (*n* = 181), 77.1% (*n* = 168) and 66.1% (*n* = 144) of all participants, respectively. Figure 1 shows the five BREQ-2 subscales.

Differences between groups are shown in Table 2.

**Table 2:** Differences between groups in BREQ-2, P-ESES-DK, and SF-36

	**EXE vs CON**								
	**GA 28 weeks**			**GA 34 weeks**			**1 year after delivery**		
	**Differences**	**95% CI**	**p**	**Differences**	**95% CI**	**p**	**Differences**	**95% CI**	**p**
**BREQ-2**									
**Amotivation**	-0.17	[-0.41; 0.04]	0.107	0.09	[-0.12; 0.27]	0.400	-0.06	[-0.33; 0.17]	0.619
**External regulation**	-0.07	[-0.27; 0.15]	0.508	0.07	[-0.11; 0.26]	0.449	-0.18	[-0.39; 0.04]	0.107
**Introjected regulation**	0.60	[0.29; 0.92]	< 0.001	0.62	[0.29; 1.00]	< 0.001	0.17	[-0.20; 0.53]	0.357
**Identified regulation**	0.63	[0.41; 0.87]	< 0.001	0.54	[0.28; 0.79]	< 0.001	0.13	[-0.18; 0.45]	0.402
**Intrinsic regulation**	0.39	[0.16; 0.64]	< 0.001	0.40	[0.16; 0.65]	< 0.001	0.02	[-0.31; 0.37]	0.902
**P-ESES-DK**									
**P-ESES-DK summary score**	6.97	[2.05; 12.02]	0.005	9.34	[3.90; 15.24]	< 0.001	-0.11	[-6.82; 6.34]	0.969
**SF-36**									
**Physical Component Summary score**	0.49	[-2.63; 3.49]	0.764	-1.16	[-4.09; 1.70]	0.419	0.04	[-2.33; 2.24]	0.546
**Mental Component Summary score**	0.39	[-2.68; 3.86]	0.821	2.99	[-0.30; 6.64]	0.085	1.89	[-0.81; 4.88]	0.169
	**MOT vs CON**								
	**GA 28 weeks**			**GA 34 weeks**			**1 year after delivery**		
	**Differences**	**95% CI**	**p**	**Differences**	**95% CI**	**p**	**Differences**	**95% CI**	**p**
**BREQ-2**									
**Amotivation**	-0.12	[-0.36; 0.11]	0.316	0.03	[-0.15; 0.20]	0.755	-0.04	[-0.29; 0.19]	0.783
**External regulation**	0.09	[-0.15; 0.32]	0.388	0.06	[-0.12; 0.27]	0.499	-0.06	[-0.29; 0.17]	0.608
**Introjected regulation**	0.65	[0.30; 0.98]	< 0.001	0.51	[0.13; 0.91]	0.007	0.32	[-0.09; 0.73]	0.136
**Identified regulation**	0.28	[0.05; 0.51]	0.017	0.35	[0.07; 0.60]	0.008	0.10	[-0.22; 0.42]	0.525
**Intrinsic regulation**	0.21	[-0.02; 0.45]	0.076	0.35	[0.10; 0.61]	0.006	0.09	[-0.24; 0.48]	0.606
**P-ESES-DK**									
**P-ESES-DK summary score**	2.13	[-2.95; 7.43]	0.379	2.70	[-2.84; 8.27]	0.357	-0.96	[-7.81; 7.75]	0.727
**SF-36**									
**Physical Component Summary score**	0.92	[-2.29; 4.01]	0.546	0.56	[-2.54; 3.48]	0.721	0.05	[-3.02; 2.74]	0.966
**Mental Component Summary score**	0.65	[-2.18; 4.08]	0.630	1.40	[-1.74; 5.06]	0.430	0.01	[-3.31; 3.03]	0.990
	**MOT vs EXE**								
	**GA 28 weeks**			**GA 34 weeks**			**1 year after delivery**		
	**Differences**	**95% CI**	**p**	**Differences**	**95% CI**	**p**	**Differences**	**95% CI**	**p**
**BREQ-2**									
**Amotivation**	0.06	[-0.10; 0.23]	0.460	-0.06	[-0.23; 0.09]	0.444	0.03	[-0.15; 0.21]	0.698
**External regulation**	0.17	[-0.02; 0.34]	0.071	-0.01	[-0.17; 0.15]	0.970	0.12	[-0.08; 0.31]	0.301
**Introjected regulation**	0.05	[-0.26; 0.35]	0.792	-0.11	[-0.40; 0.15]	0.436	0.15	[-0.22; 0.50]	0.442
**Identified regulation**	-0.34	[-0.54; -0.13]	0.001	-0.19	[-0.39; 0.02]	0.074	-0.03	[-0.29; 0.21]	0.761
**Intrinsic regulation**	-0.18	[-0.37; 0.02]	0.068	-0.05	[-0.26; 0.14]	0.593	0.07	[-0.22; 0.32]	0.636
**P-ESES-DK**									
**P-ESES-DK summary score**	-4.83	[-9.19; -0.23]	0.041	-6.64	[-11.16; -2.14]	0.007	-0.84	[-6.24; 4.54]	0.790
**SF-36**									
**Physical Component Summary score**	-0.56	[-3.23; 1.86]	0.647	-1.31	[-3.82; 0.99]	0.256	-0.78	[-3.31; 1.27]	0.488
**Mental Component Summary score**	-1.62	[-3.93; 0.84]	0.239	-0.57	[-2.70; 1.71]	0.670	-0.29	[-2.67; 2.08]	0.781

Differences (estimates, 95% CI and *p*-values) between groups in the subscales of Behavioural Regulation in Exercise Questionnaire-2, the summary score of the Pregnancy Exercise Self-Efficacy Scale, and the Physical and Mental Component Summary scores of the Short Form 36 Health Survey Questionnaire at GA 28 weeks, GA 34 weeks and one year after delivery. *GA* gestational age in weeks, *EXE* structured supervised exercise training, *MOT* motivational counselling on physical activity, *CON* standard prenatal care

Participants in EXE and MOT had a higher introjected and identified regulation mean score compared to CON at GA 28 weeks and GA 34 weeks. The intrinsic regulation at GA 28 weeks was higher in EXE compared to CON, and in EXE and MOT compared to CON at GA 34 weeks. MOT had lower identified regulation at GA 28 weeks compared to EXE. One year after delivery, there were no differences between groups in any of the BREQ-2 subscales, and across groups the outcomes remained unchanged compared to the baseline tests. Observations of exercise behavioural regulation throughout the study period are visualised in Fig. 1 and reported descriptively in Table 3.

**Table 3:** Descriptive observations of BREQ-2, P-ESES-DK, and SF-36 throughout the study period

	**All**	**EXE**		
	**Common baseline**	**GA 28 weeks**	**GA 34 weeks**	**1 year after delivery**
**BREQ-2**				
**Amotivation**	0.30 [0.24; 0.38]	0.24 [0.14; 0.36]	0.33 [0.21; 0.47]	0.27 [0.15; 0.41]
**External regulation**	0.56 [0.47; 0.65]	0.48 [0.37; 0.61]	0.47 [0.36; 0.59]	0.31 [0.20; 0.45]
**Introjected regulation**	1.88 [1.75; 2.00]	1.98 [1.76; 2.19]	1.97 [1.77; 2.17]	1.63 [1.38; 1.88]
**Identified regulation**	2.34 [2.24; 2.45]	2.65 [2.47; 2.82]	2.68 [2.53; 2.84]	2.35 [2.14; 2.55]
**Intrinsic regulation**	2.70 [2.58; 2.81]	2.77 [2.60; 2.92]	2.76 [2.60; 2.91]	2.51 [2.29; 2.71]
**P-ESES-DK**				
**P-ESES-DK summary score**	36.71 [25.85; 37.55]	31.49 [28.51; 34.62]	30.37 [27.25; 33.61]	22.14 [18.58; 26.16]
**SF-36**				
**Physical Component Summary score**	53.29 [52.44; 54.14]	46.63 [44.68; 48.49]	43.22 [41.54; 44.92]	54.96 [53.00; 56.49]
**Mental Component Summary score**	50.12 [49.20; 51.11]	54.02 [52.07; 55.69]	55.69 [54.20; 57.05]	52.71 [51.24; 54.16]
		**MOT**		
		**GA 28 weeks**	**GA 34 weeks**	**1 year after delivery**
**BREQ-2**				
**Amotivation**		0.29 [0.18; 0.41]	0.27 [0.17; 0.38]	0.30 [0.18; 0.43]
**External regulation**		0.65 [0.51; 0.80]	0.46 [0.36; 0.59]	0.43 [0.27; 0.61]
**Introjected regulation**		2.03 [1.79; 2.26]	1.86 [1.63; 2.08]	1.79 [1.49; 2.08]
**Identified regulation**		2.30 [2.13; 2.45]	2.48 [2.32; 2.66]	2.32 [2.10; 2.51]
**Intrinsic regulation**		2.59 [2.43; 2.75]	2.71 [2.54; 2.87]	2.58 [2.36; 2.81]
**P-ESES-DK**				
**P-ESES-DK summary score**		26.66 [23.31; 29.62]	23.73 [20.31; 27.00]	21.30 [17.68; 25.26]
**SF-36**				
**Physical Component Summary score**		47.05 [45.07; 49.00]	44.95 [43.03; 46.86]	54.98 [52.47; 56.98]
**Mental Component Summary score**		54.27 [52.49; 56.10]	54.10 [52.26; 55.95]	50.83 [48.63; 52.86]
		**CON**		
		**GA 28 weeks**	**GA 34 weeks**	**1 year after delivery**
**BREQ-2**				
**Amotivation**		0.41 [0.22; 0.61]	0.24 [0.10; 0.41]	0.34 [0.14; 0.56]
**External regulation**		0.56 [0.37; 0.73]	0.41 [0.24; 0.57]	0.49 [0.32; 0.67]
**Introjected regulation**		1.38 [1.13; 1.64]	1.35 [1.03; 1.64]	1.47 [1.17; 1.77]
**Identified regulation**		2.02 [1.81; 2.23]	2.14 [1.91; 2.35]	2.22 [1.95; 2.49]
**Intrinsic regulation**		2.37 [2.15; 2.58]	2.36 [2.12; 2.58]	2.49 [2.18; 2.78]
**P-ESES-DK**				
**P-ESES-DK summary score**		24.52 [20.49; 28.47]	21.03 [16.32; 25.74]	22.26 [17.23; 27,60]
**SF-36**				
**Physical Component Summary score**		46.14 [43.62; 48.68]	44.39 [42.04; 46.89]	54.92 [53.30; 56.49]
**Mental Component Summary score**		53.62 [50.43; 56.00]	52.70 [49.44; 55.69]	50.83 [48.37; 53.04]

Descriptive observations (estimates, 95% CI and *p*-values) of the subscales of Behavioural Regulation in Exercise Questionnaire-2, the summary score of the Pregnancy Exercise Self-Efficacy Scale, and the Physical and Mental Component Summary scores of the Short Form 36 Health Survey Questionnaire at GA 28 weeks, GA 34 weeks and one year after delivery. *GA* gestational age in weeks, *EXE* structured supervised exercise training, *MOT* motivational counselling on physical activity, *CON* standard prenatal care

### Pregnancy exercise self-efficacy

**Fig. 2:**
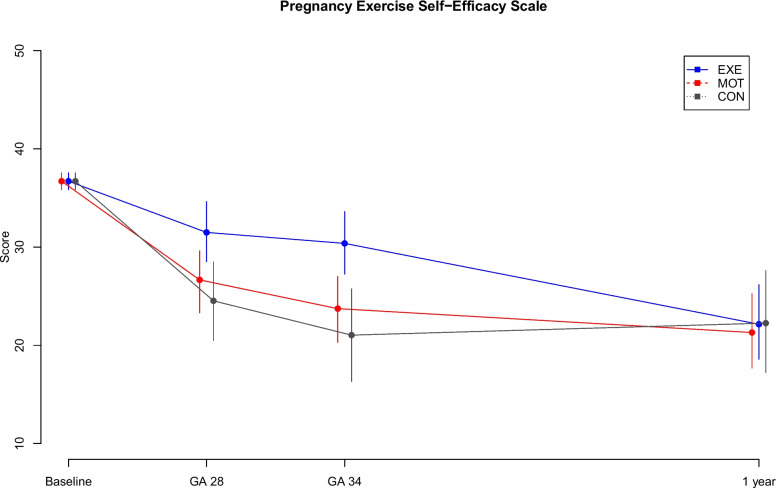
Pregnancy exercise self-efficacy scale The summary score of the Danish version of the Pregnancy Exercise Self-Efficacy Scale (mean and 95% CI) measured at baseline, GA 28 weeks, GA 34 weeks and one year after delivery. GA, gestational age in weeks; blue, structured supervised exercise training (EXE); red, motivational counselling on physical activity (MOT); grey, standard prenatal care (CON)

P-ESES-DK was completed at baseline, GA 28 weeks, GA 34 weeks, and one year after delivery by 100% (*n* = 218/218), 83.0% (*n* = 181), 77.1% (*n* = 168) and 62.8% (*n* = 137) of all participants, respectively. Figure 2 shows the summed score of the 10 P-ESES-DK items. At baseline, all participants on average indicated a high exercise self-efficacy score, and all group estimates decreased during pregnancy and one year after delivery indicating a medium exercise self-efficacy (Fig. 2).

Mean score differences between groups are shown in Table 2. The total exercise self-efficacy was higher in EXE compared to both CON and MOT at GA 28 and 34 weeks. Observations of exercise self-efficacy throughout the study period are visualised in Fig. 2 and reported descriptively in Table 3. The most prominent decline in exercise self-efficacy was observed among participants in MOT and CON during pregnancy.

Additional file 1 shows the differences between groups in each of the 10 P-ESES-DK items. Participants in EXE had significantly higher exercise self-efficacy than participants in CON in item 1–6 at GA 28 and 34 weeks and in item 9 at GA 28 weeks. Participants in MOT had lower exercise self-efficacy than participants in EXE in item 1, 4, 5 and 6 at GA 28 and 34 weeks and in item 3 at GA 34 weeks.

### Health-related quality of life

**Fig. 3:**
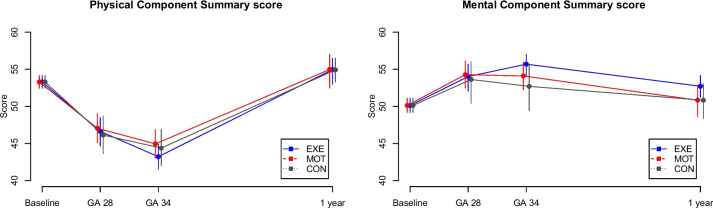
The Short Form 36 Health Survey Questionnaire Physical and Mental Component Summary scores The Short Form 36 Health Survey Questionnaire Physical and Mental Component Summary scores (mean and 95% CI) measured at baseline, GA 28 weeks, GA 34 weeks and one year after delivery. GA, gestational age in weeks; blue, structured supervised exercise training (EXE); red, motivational counselling on physical activity (MOT); grey, standard prenatal care (CON)

SF-36 was completed at baseline, GA 28 weeks, GA 34 weeks, and one year after delivery by 99.5% (*n* = 217/218), 83.0% (*n* = 181), 77.5% (*n* = 169) and 66.1% (*n* = 144) of all participants, respectively. Figure 3 shows the Physical and Mental Component Summary scores, respectively.

No differences were found between groups in the two component summary scores, neither during pregnancy nor one year after delivery (Table 2). However, EXE tended to have a higher Mental Component Summary score at GA 34 weeks compared to CON. Observations of HRQoL throughout the study period are visualised in Fig. 3 and reported descriptively in Table 3. For all participants, the Physical Component Summary score declined during pregnancy and reached just above the baseline value one year after delivery. In contrast, the Mental Component Summary score increased during pregnancy and reached just above the baseline value one year after delivery.

The associations between PA and the psychosocial factors are presented as Additional file 2. In EXE, higher moderate-to-vigorous-intensity PA was associated with higher scores of BREQ Introjected, BREQ Identified, and BREQ Intrinsic, and P-ESES-DK. In MOT, higher moderate-to-vigorous-intensity PA was associated with higher scores of BREQ Extrinsic and with lower scores of Mental Component Summary. In CON, higher moderate-to-vigorous-intensity PA was associated with higher scores of BREQ Identified and P-ESES-DK.

### Sickness absence and sick leave

Table 4 shows the level of and reasons for sickness absence and sick leave at inclusion and at GA 28 weeks. At GA 28 weeks, sickness absence and sick leave were equally distributed between the three groups (sickness absence: EXE: 28.7%, *n* = 25/87; MOT: 24.4%, *n* = 21/86; CON: 26.7%, *n* = 12/45, *p* = 0.807; sick leave: EXE: 24.1%, *n* = 21/87; MOT: 16.3%, *n* = 14/86; CON: 11.1%, *n* = 5/45, *p* = 0.297), which also applied when sick leave in EXE and MOT were compared to CON (*p* = 0.160 and *p* = 0.554, respectively). No differences were found between groups in reasons for sickness absence or sick leave (*p* = 0.905 and *p* = 0.668, respectively), nor for part-time leave compared to full-time leave (*p* = 0.797).

**Table 4:** Sickness absence and sick leave

	**Inclusion**		**GA 28 weeks**	
	**Sickness absence**	**Sick leave**	**Sickness absence**	**Sick leave**
	*n* = 65	*n* = 21	*n* = 58	*n* = 40
Number of days				
0–4	41 (65.1%)	3 (14.3%)	36 (62.0%)	4 (10.0%)
5–8	13 (20.0%)	2 (9.5%)	18 (31.0%)	3 (7.5%)
9–14	6 (9.2%)	1 (4.8%)	3 (5.2%)	3 (7.5%)
> 14	5 (7.7%)	15 (71.4%)	1 (1.7%)	30 (75.0%)
Category of sick leave		^a^		^c^
Part-time	No data	12 (57.1%)	No data	14 (37.8%)
Full-time	No data	3 (14.3%)	No data	23 (62.1%)
Reasons	^b^		^d^	
Pregnancy	36 (55.4%)	13 (61.9%)	27 (46.6%)	33 (82.8%)
Non-pregnancy	28 (43.1%)	4 (19.0%)	29 (50.0%)	6 (15.0%)
Environment	0 (0%)	4 (19.0%)	0 (0%)	1 (2.5%)

Number and percentages (%) for sickness absence and sick leave at inclusion and at GA 28 weeks

*GA* gestational age in weeks

^a^ six missing values

^b^ one missing value

^c^ three missing values

^d^ two missing values

Additional file 3 presents the prevalence of low back and pelvic pain and the intensity of pain on group level.

### Low back and pelvic girdle pain

Table 5 shows the prevalence (daily, weekly, or never) and the intensity of pain (if pain is experienced daily or weekly) when the pain is experienced as lowest, highest and at average during a day. At GA 28 weeks, no differences were found between groups in regard to prevalence (EXE: 67.8%, *n* = 59/87; MOT: 68.6%, *n* = 59/86; CON: 53.3%, *n* = 24/45, p = 0.664) or intensity of pain (e.g. intensity of pain on average in EXE, MOT and CON: 3 (2–4), Additional file 3).

**Table 5:** Low back and pelvic girdle pain

	**Prior to pregnancy**	**Inclusion**	**GA 28 weeks**
	*n* = 218	*n* = 218	*n* = 180
Low back and pelvic girdle pain			
Daily	11 (5.0%)	43 (19.8%)	78 (43.3%)
Weekly	29 (13.3%)	58 (26.6%)	64 (35.6%)
Never	178 (81.7%)	117 (53.7%)	38 (21.1%)
Intensity of pain			
Lowest	0 (0–1)	0 (0–1)	0 (0–1)
Highest	5 (4–7)	5 (3–7)	6 (4–7)
On average	2 (2–3)	2 (1–3)	3 (2–4)

Number, percentages (%) and median (IQR) for low back and pelvic girdle pain prior to pregnancy, at inclusion and at GA 28 weeks

*GA* gestational age in weeks

## Discussion

This study highlights that structured supervised exercise training and motivational counselling on PA positively influenced exercise behavioural regulation and self-efficacy in pregnant women. However, the positive effects were not maintained in a long term. The study did not find significant effects on HRQoL, sickness absence, or pain.

Both EXE and MOT interventions increased autonomous motivation for exercise compared to CON during pregnancy. In general as well as among pregnant women, autonomous motivation is associated with positive changes in PA level and maintenance [53, 54], but in the FitMum trial presented by Knudsen et al. [36], only participants in EXE had higher moderate-to-vigorous-intensity PA during pregnancy compared to CON. The two interventions represent targeted and organised interventions. This structure may have contributed to enhancing participant autonomy by providing clear guidelines and goals for their PA. This approach may increase motivation as the participants feel more supported and informed compared to participants in CON.

Moreover, the present study found that participants in EXE, and not in MOT, had higher exercise self-efficacy during pregnancy compared to participants in CON. Thus, exercise self-efficacy appears to be maintained most effectively during pregnancy with a supervised PA intervention. Interestingly, exercise behavioural regulation was increased with both supervised and non-supervised interventions, while structured exercise training was pivotal for enhancing exercise self-efficacy. This might be attributed to the structured and supervised nature of the EXE intervention providing clear and consistent support with skill development fostering participants' confidence. The group dynamics in the sessions may have created a supportive environment, enhancing the participants' belief in their exercise capabilities. Lastly, while motivational counselling in MOT may have increased the PA motivation, it might not have been as effective in building the practical skills crucial for exercise, potentially leading to lower exercise self-efficacy compared to EXE. These findings are important to note when exercise is offered to pregnant women in future interventions to increase their PA level. As with autonomous motivation, a previous study suggested that exercise self-efficacy is associated with higher levels of PA [21], which is in line with the present findings. The long-term data on exercise behavioural regulation and exercise self-efficacy one year after delivery may indicate that differences between groups during pregnancy level out when participants are no longer exposed to the interventions. The exercise self-efficacy data reported one year after delivery should be interpreted with caution as the questionnaire is developed to obtain data during pregnancy.

HRQoL differences between groups were minor, except for EXE potentially having higher Mental Component Summary score late in pregnancy compared to CON. Somewhat in line with this, a systematic review suggested that high-frequency group-based aerobic and resistance training during pregnancy are beneficial for improving pregnant women’s HRQoL [55]. The results of the Physical and Mental Component Summary scores across groups in the present study are consistent with results from a systematic review on factors influencing the HRQoL of pregnant women [56]. The review found that pregnant women, especially in late pregnancy, have significantly lower physical HRQoL compared to non-pregnant women of the same age, whereas the score of the mental part of HRQoL of the pregnant women increased or remained stable over the course of the trimesters. It is well known that PA is positively associated HRQoL among the general adult population [57]. However, pregnancy appears to exert a significant influence, as we do not observe the same correlation within this specific population. The complex interplay of factors during pregnancy may contribute to variations in the relationship between PA and HRQoL, highlighting the need for a nuanced understanding of these dynamics in pregnant women. The lack of effects on HRQoL in the present study may also be a result of other issues: the generic questionnaire SF-36 may not be sensitive enough to detect differences between the included groups, the sample size of participants may be too low to detect differences, and HRQoL of the participants may simply not be affected by the performed exercise [58].Notably, no significant group differences emerged in sickness absence or musculoskeletal pain. This may stem from very limited reports of these issues at baseline. Consequently, detecting any noticeable effects on absence and pain became challenging due to the concentration of responses at the lowest end of the measurement scale. The present study found a higher rate of sickness absence (sickness absence and sick leave at GA 28 weeks: 26.6% and 18.3%, respectively) compared to studies from 2011–17 [59], which reported that 13.5% of Danish women had reported sickness absence (with or without a medical certificate) during their pregnancy. The prevalence of pregnancy-related low back pain has in another study been estimated to be above 50% [60], which corresponds to the findings in the present study (pain at GA 28 weeks: daily 43.3%, weekly: 26.6%). Additionally, the participants reported a relatively high intensity of pain when they were asked about the highest intensity level experienced, which may impact their ability to perform PA in general.

This study holds significant and important clinical and public health implications. The examination of the interventions’ effects on PA behaviour and psychosocial factors aimed to deepen our understanding of how the interventions influence both PA levels and personal mental capacity to become more physically active. Notably, supervision in exercise interventions seemed to exert a more pronounced impact on the exercise behavioural regulation and exercise self-efficacy compared to a purely motivational approach lacking the supervised physical guidance. Consequently, when planning interventions targeting pregnant women with low PA levels, considering supervised programs becomes vital to enhance both PA engagement and psychosocial aspects linked to PA. In addition, clinicians must be aware of the pivotal role of clear supervision and guidance and continuous support in pregnant women's PA motivation and self-efficacy.Strengths and limitations.

The randomised design of the FitMum trial is a strength and enabled comparisons between groups, even though the present study was not powered to analyse the effects on the present secondary outcomes. Combining several PA dimensions through questionnaires provided insight into the complexity of PA behaviour, and the self-administration and low cost of the measurements enabled repeated measurements during the trial with only minor inconvenience for the participants. However, the inherent bias of self-reporting data is inevitable. The requirement to recall one’s PA motives and behaviour may be difficult, especially in a transformational period of life as pregnancy.

BREQ-2 and P-ESES are grounded in behaviour change sciences [19, 22], and the Danish version of P-ESES (P-ESES-DK) used is valid and reliable [40], which strengthen the quality of the study. A limitation is that BREQ-2 and SF-36 were developed without any adaptation to the unique experiences of pregnancy period and the year after delivery. Although there were studies reporting the adequacy of these measures in diverse populations, their adequacy for pregnant and postnatal populations has not been established.

## Conclusion

This secondary analysis of the FitMum trial showed that prenatal structured supervised exercise training and motivational counselling on PA increased autonomous motivation for PA in pregnant women compared with standard prenatal care. Further, participation in structured supervised exercise training, but not in motivational counselling on PA, increased exercise self-efficacy compared to standard prenatal care. There were no differences between groups when examining HRQoL, sickness absence, and low back and/or pelvic girdle pain during pregnancy. Importantly, the positive findings disappeared one year after delivery when the interventions were discontinued. This may emphasise the importance of continuous PA interventions after delivery to maintain the motivation for PA.

### Supplementary Information

**Additional file 1: **Differences between groups in all items of the Danish version of the Pregnancy Exercise Self-Efficacy Scale.

**Additional file 2: **Associations between moderate-to-vigorous intensity physical activity and psychosocial factors.

**Additional file 3: **Sickness absence, sick leave and low back and pelvic pain on group level at gestational age 28 weeks.

## Data Availability

The datasets used in the present study are not publicly available due to confidentiality but are available from the corresponding author on reasonable request. We can transfer individual participant data when we have obtained approval from the Danish Data Protection Authority according to the Data Protection Act and completed a Standard Contractual Clause to ensure the legal basis of the transfer.

## Abbreviations

BREQ-2: Behavioural Regulation in Exercise Questionnaire-2
CON: Standard prenatal care
EXE: Structured supervised exercise training
GA: Gestational age
HRQoL: Health-related quality of life
MOT: Motivational counselling on physical activity
PA: Physical activity
P-ESES-DK: The Danish version of the Pregnancy Exercise Self-Efficacy Scale
SF-36: Short Form 36 Health Survey Questionnaire
